# A Strategy to Enhance the Electrode Performance of Novel Three-Dimensional PEDOT/RVC Composites by Electrochemical Deposition Method

**DOI:** 10.3390/polym9050157

**Published:** 2017-04-28

**Authors:** Ali Aldalbahi, Mostafizur Rahaman, Mohammed Almoiqli

**Affiliations:** 1 Department of Chemistry, College of Science, King Saud University, Riyadh 11451, Saudi Arabia; mrahaman@ksu.edu.sa; 2 Nuclear Sciences Research Institute, King Abdulaziz City for Science and Technology, Riyadh 12371, Saudi Arabia; almoiqli@kacst.edu.sa

**Keywords:** PEDOT/RVC composites, electrochemical deposition, electrode performance, morphology, thermal stability, cyclic voltammetry

## Abstract

In this article, three-dimensional (3D) microstuctured poly(3,4-ethylenedioxythiophene) (PEDOT)/reticulated vitreous carbon (RVC) composite electrodes with varying amount of PEDOT loadings were successfully prepared by electrochemical deposition method. The composites were characterized by Raman spectroscopy, thermogravimetric analysis (TGA), scanning electron microscopy (SEM), and cyclic voltammetry. Raman spectra suggest that there is a strong interaction between the RVC and backbone of PEDOT chain. It is revealed from the SEM images that the PEDOT amount, thickness, surface roughness, porosity, and globular structure on RVC electrode are increased with the increase in polymerization time. The capacitance of PEDOT/RVC electrode has increased by a factor of 2230 compared to a bare RVC electrode when polymerization is carried out for 120 min. Moreover, the capacitance of PEDOT was found to be very high compared with other PEDOT studies. The electrodes also show good cyclic stability. This substantial increase in capacitance of RVC electrode is due to the rough, highly porous, and honeycomb-like fine structure of PEDOT coating, which shows a flower-like morphology, consisting of numerous thin flakes with numbers of macropores and micropores. This interesting morphology has enhanced the performance of PEDOT because of increased electrode surface area, specific capacitance, and macroporous structure of RVC electrode.

## 1. Introduction

Nowadays, many researchers have focused on the development of novel carbon materials [1,2,3,4,5,6,7,8,9] such as activated carbon, carbon aerogel, carbon nanotubes, graphene, ordered mesoporous carbon, and their composites to make electrodes with high specific surface area, high conductivity, reasonable microstructured pores, and high electrosorption capacity. In addition, conducting polymers have been combined with such carbon materials to improve the carbon electrode properties and performance [10,11,12] because they have high electrical conductivity and high surface area [13,14]. All these electrodes were built as two-dimensional structure electrodes without significant thickness. However, a limited improvement in the electrosorption capacity is still far less than the theoretical value, which is mainly due to shortcomings such as the low effective surface area [15].

The conducting polymer PEDOT (poly(3,4-ethylenedioxythiophene)) is an active material for the preparation of an electrode because of its interesting properties such as high electrical conductivity, surface area, environmental stability [16], facile synthesis via electrochemical polymerization, etc. In addition, a novel PEDOT material can be used as a three-dimensional porous electrode with well-interconnected macropores, and numerous mesopores and micropores embedded in the reticulated vitreous carbon (RVC) walls because this improves ion diffusion by providing a decreased ion diffusion distance [15]. PEDOT has also been used in bioelectronics for coating on medical devices and in making highly performed electrochemical transistor [17,18]. The presence of macropores, serving as ion-buffering reservoirs, guarantees a shorter ion diffusion distance [19,20], which facilitates the rapid transportation of the ions into the interior of the bulk material. Moreover, it has been demonstrated that 3D porous carbon with an interconnected pore system shows an excellent performance in the field of electrochemistry [20,21,22,23,24,25].

RVC as a substrate was used for PEDOT film deposition because it has three-dimensional (3D) porous structure that provides high macroscopic surface area and other interesting characteristics such as a low density, high chemical inertness with low electrical and fluid flow resistance, and is also amenable to surface modification with conducting polymers [26,27,28,29].

This article describes and discusses the electrodeposition of various amounts of PEDOT to coat RVC electrodes. These electrodes were characterized for their morphology, Raman spectrum, thermal stability, and electrochemical properties.

## 2. Materials, Methods, and Experimental

### 2.1. Chemicals and Materials

Commercial 3,4-ethylenedioxythiophene monomer (EDOT) with purity 99.9% was purchased from Sheng Chemical Ltd. (Taichung, Taiwan), and was used as received. The following chemicals, obtained from Sigma-Aldrich (Darmstadt, Germany), were also used as received: acetonitrile (ACN) (AR grade), lithium perchlorate (LiClO_4_) (AR grade), concentrated nitric acid (70%) and sodium chloride (AR grade). The reticulated vitreous carbon (RVC) (60 ppi (normal pores per linear inch)) was purchased from ERG Materials and Aerospace Engineering and used as received. Milli-Q water with a resistivity of 18.2 mΩ cm^−1^ was used in all preparations.

### 2.2. Pre-Treatment of the RVC Electrode

All reticulated vitreous carbon (RVC) electrodes (length 4 cm × width 3.5 cm × thickness 0.3 cm (32.5 or 4.2 cm^3^) were cut from a block of RVC material, and soaked in 2 M HNO_3_ for 24 h to remove any impurities [30]. Electrodes were thoroughly washed with distilled water to remove the acid. The pH of the effluent was checked periodically until the wash became neutral. All RVC electrodes were then soaked in methanol for 2 h to remove any organic impurities [30]. The RVC electrodes were dried under a brief flow of nitrogen and kept in an oven at 110 °C overnight. All RVC electrodes were weighed after drying.

### 2.3. Electrochemical Polymerization of PEDOT on RVC Electrode

In this work, the PEDOT/RVC composite electrodes were synthesized by cyclic voltammetric and chronoamperometry. In both techniques before polymerizations, the RVC pieces were left in contact with the working electrolyte for at least 24 h to ensure their complete wetting. In order to prepare the working electrodes, electrical contact was made by a hook of Pt wire. The electropolymerization was performed in an organic electrolyte, where the reference electrode was an Ag/AgCl (3 M NaCl) electrode and the counter electrode was a Pt mesh with size 4 × 4 cm^2^. The electrolyte was an acetonitrile solution containing 0.01 M monomer, EDOT and 0.1 M supporting electrolyte salt (LiClO_4_) [31,32]. The solution was thoroughly deoxygenated by nitrogen (N_2_) before the electropolymerization for 10 min prior to all electrochemical experiments at room temperature. PEDOT was deposited on the RVC working electrode by cyclic voltammetry using three-electrode systems in the voltage range between 0 and 1.3 V at 50 mV/s scan rate. In addition, PEDOT films were galvanostatically deposited on the RVC working electrode with a constant voltage applied for various periods of time. The quantity of PEDOT-ClO_4_ coating on the RVC electrodes was determined by calculating the total charge passed in the electropolymerization. The charge values were read directly from the I to V curves by computer.

### 2.4. Physical Characterization

The thermal stability of the PEDOT on PEDOT/RVC electrodes and the amount of PEDOT coated on the RVC were determined by thermogravimetric analysis (TGA). Experiments were performed using a Q500 (TA Instruments, New Castle, DE, USA) apparatus at a ramp rate of 5 °C/min in air, with a combined gas flow of 10 mL min^−1^ Nitrogen (N_2_) and 90 mL min^−1^ air from 25 to 750 °C. Moreover, the morphology and the thickness of the PEDOT deposits on RVC electrodes were analyzed by using a field emission scanning electron microscope (FESEM, ZEISS Sigma, Hamburg; Germany) at specific voltages of 0.5 KV. Furthermore, Raman spectra were measured on a Raman spectrometer equipped with a visible Raman microscope and CCD detector. The excitation wavelength was 632.81 nm and spectra were obtained over 30 s at 1.0 cm^−1^ resolution.

### 2.5. Electrochemical Characterization

The capacitance and the effect of different scan rates were determined by cyclic voltammetry (CV). A PEDOT/RVC composite electrode was used as the working electrode (WE) in 1 M NaCl aqueous solution and scanned in the voltage range between −0.2 to 0.8 V using a three-electrode system; RVC electrode and Ag/AgCl (3 M NaCl) were used as counter electrode (CE) and reference electrode (RE), respectively. The scan rates range from 5 to 200 mV/s. Contacts to the WE and CE were made using Pt wire.

## 3. Results and Discussion

### 3.1. PEDOT Deposited on RVC Electrode

PEDOT can be polymerized using multiple different ways, but for the purposes of this work, electrochemical polymerization has been used to synthesize PEDOT. This method is important because it requires only a small amount of monomer, short polymerization time, and can yield both electrode-supported and free-standing film. This method utilizes electrochemical oxidation of the electron-rich EDOT-based monomers by three different techniques; namely, cyclic voltammetry, chronoamperometry and chronopotentiometry. In this project, chronoamperometry method was selected to grow thick PEDOT coated RVC electrodes and cyclic voltammetry was used to select the potential of EDOT monomer oxidation to form PEDOT.

### 3.2. Cyclic Voltammetry

**Figure 1:**
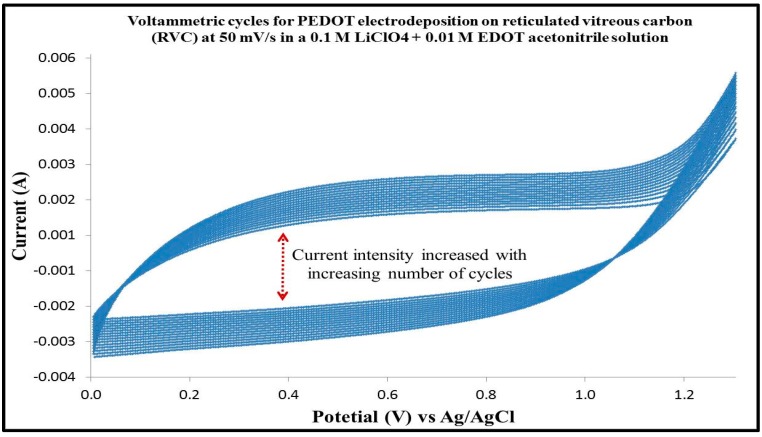
Cyclic voltammetry of RVC electrode in a solution containing 0.01 M EDOT and 0.1 M LiClO_4_ in acetonitrile, using a three-electrode system; RVC electrode and Ag/AgCl (3 M NaCl) were used as counter electrode (CE) and reference electrode (RE), respectively.

EDOT monomer was prepared in acetonitrile as electrolyte because it has the advantage of a higher conductivity than that prepared in aqueous solution [33]. Figure 1 shows the cyclic voltammograms related to the EDOT. It can be seen from the figure that the EDOT oxidation started at 1.1 V for the anodic scan. Furthermore, the EDOT electropolymerization is characterized by cycles with a crossover of the reverse cathodic scan over the anodic scan, giving rise to what has been called the “nucleation loop” [34]. In particular, we observe that the current of the reverse scan is higher than that of the forward scan in the region close to the switching potential, but it drops again to the level of the current–voltage curve of the forward scan. Such kind of CV profiles have been interpreted as due to polymer nucleation effects [35] or, more recently, to homogeneous reactions between an oligomeric follow-up product and the starting monomer [36]. Moreover, an increase of both anodic and cathodic current intensities with increasing number of scans is also evident. This effect can be explained by considering the growth at the electrode surface of an electroactive polymer film, whose thickness increases regularly with the number of cycles [37]. Moreover, the RVC foam electrode was observed after polymerization to have a visible bluish color on the foam skeleton and this has been reported in available literature [38]. The polymerization mechanism of PEDOT has been described in Supplementary Materials.

### 3.3. Effect of Applied Constant Potential on PEDOT Electrosynthesis

**Figure 2:**
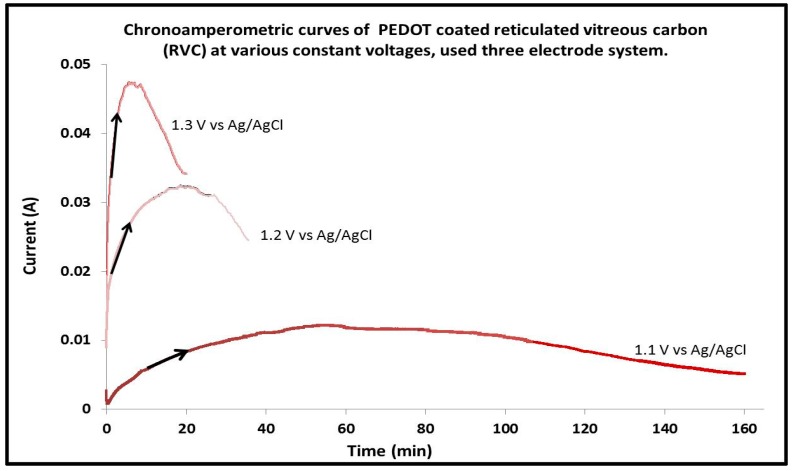
Chronoamperometric curves at various constant potential applied to coat RVC electrodes by PEDOT in a solution containing 0.01 M EDOT and 0.1 M LiClO_4_ in acetonitrile, using a three-electrode system; RVC electrode and Ag/AgCl (3 M NaCl) were used as counter electrode (CE) and reference electrode (RE), respectively.

Chronoamperometry was used to deposit PEDOT on RVC electrodes. The aim of this experiment is to study the effect of increasing the constant potential on electrochemical polymerization and to determine the best constant potential to be used for further experiments. These potentials were selected based on the CV of PEDOT growth (Figure 1) obtained previously that showed no over-oxidation of the polymer because polymer growth continued to increase with increasing number of cycles. Current–time curves for the electrodeposition of EDOT by potential steps 1.1, 1.2 and 1.3 V are shown in Figure 2. All of these curves attain to the same charge consumed of 48 C. It is clear that the general features of these curves are similar to those reported in the literature [39,40,41]. The current starts to increase because the molecules of monomer diffuse from the solution to the electrode surface and, once they are oxidized, return to the solution where the oligomerization process occurs in the vicinity of the electrode surface. When an oligomeric high density region is established, clusters are deposited onto the electrode creating the growing nuclei. After that, the current increases until it reaches a current plateau. This region is generally attributed to nucleation and growth. The phenomena of nucleation and growth of PEDOT were also observed and reported in published literature [42]. Figure 2 also shows that PEDOT electrodeposition on RVC at a constant voltage of 1.1 V was too slow and it took time (around 160 min) to attain a charge consumed of 48 C. It can be observed that when the constant potential applied was increased from 1.1 to 1.2 and 1.3 V, the current increased as expected. This led to a decrease in the time required to electrodeposit PEDOT on the RVC. For example, the same RVC electrode at a constant potential of 1.3 V required 20 min to attain the same charge consumed of 48 C. It is clear that the polymerization time decreased 8 times at 1.3 V compared with that at 1.1 V. In addition, when the potential of deposition is increased, the porosity of PEDOT surface morphology is increased [43]. According to these reasons, the constant voltage of 1.3 V was selected as the optimum potential to deposit PEDOT on RVC electrode using the potentiostatic mode in the work presented earlier.

### 3.4. Electrodeposition of Different Amounts of PEDOT on RVC Electrodes

**Figure 3:**
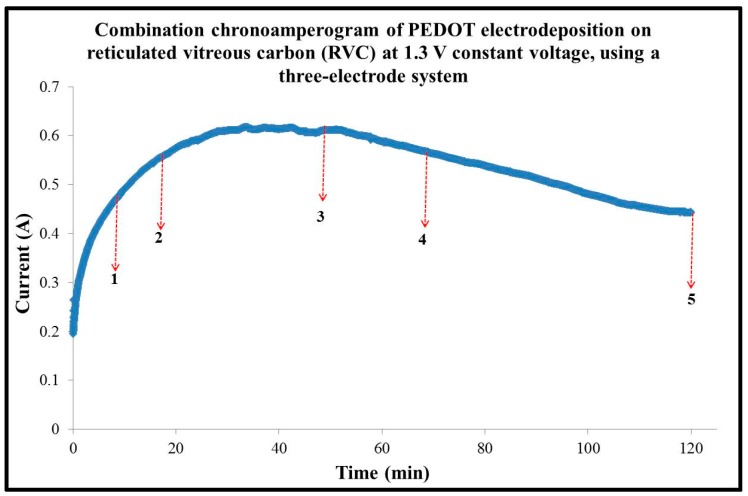
Combination chronoamperogram at 1.3 V constant potential obtained at RVC electrodes (4.2 cm^3^) in a solution containing 0.01 M EDOT and 0.1 M LiClO_4_ in acetonitrile, using a three-electrode system; RVC electrode and Ag/AgCl (3 M NaCl) were used as counter electrode (CE) and reference electrode (RE), respectively (arrows with number 1, 2, 3, 4, and 5 indicate the polymerization time for each electrode).

The PEDOT films were electropolymerized onto (4 × 3.5 × 0.3 cm^3^) RVC electrodes by applying a constant potential of 1.3 V vs. Ag/AgCl until polymerization times of 10, 20, 50, 70 and 120 min were reached. The aim of these experiments is to increase the amount of PEDOT on the RVC. Figure 3 shows a combination of chronoamperometric curves of PEDOT deposition on RVC electrodes and the arrows indicate the polymerization times for each electrode. It is clear that the chronoamperogram started at 0.19 A, then immediately rose sharply to 0.32 A within the first 5 min. After that, the curve increased dramatically to reach 0.62 A after 35 min. The chronoamperometric curve plateaued for 18 min then started to decrease steadily to 0.46 A when finally the current became almost stable. The charge consumed at each electrode was read directly from the I to V curve by computer and it was 20.64, 46.04, 112.71, 185.73 and 380.98 Coulombs (C) for polymerization times 10, 20, 50, 70 and 120 min, respectively. Table 1 shows the polymerization time, charge passed through the electrode, and the mass of PEDOT coating on the RVC electrode calculated using the following Equation (1) [44]:

(1) $$m=\frac{\left(Q\;\times \;M_{\mathrm{EDOT}}\right)\;+\left(Q\;\times \gamma \times M_{{\mathrm{ClO}}_{4}}\;\right)}{n\;\times \;F}$$

where, *m* is mass (g); $M_{\mathrm{EDOT}}$ is molecular weight (142.16 g/mol) of EDOT; $M_{{\mathrm{ClO}}_{4}}\;$ is molecular weight (99.45 g/mol) of ClO_4_; Q is charge passed (C) on the working electrode; *n* is the number of electrons transferred, which equals to 2 + γ, where two electrons are associated with polymerization and γ electrons are associated with doping of one monomer unit in PEDOT; and F is the faraday constant (96,485.34 C). The doping level γ can be determined from the relation *Q*_max_ *=* (γ/(2 + γ))*Q*, where *Q*_max_ is the charge density at the maximum quantity of oxidized polymer [45]. The value of *Q*_max_ is calculated by integrating the current from its initial potential to the potential just after anodic peak. The plot of *Q*_max_ vs. *Q* gives a linear line with the slope γ/(2 *+* γ). This results γ = 0.45, and hence the value of *n* = 2.45. It is seen from the table that the PEDOT mass in each electrode increased with increase in polymerization time. It was 13, 29, 71, 117 and 240 mg for polymerization times 10, 20, 50, 70 and 120 min, respectively.

### 3.5. PEDOT Surface Properties

**Figure 4:**
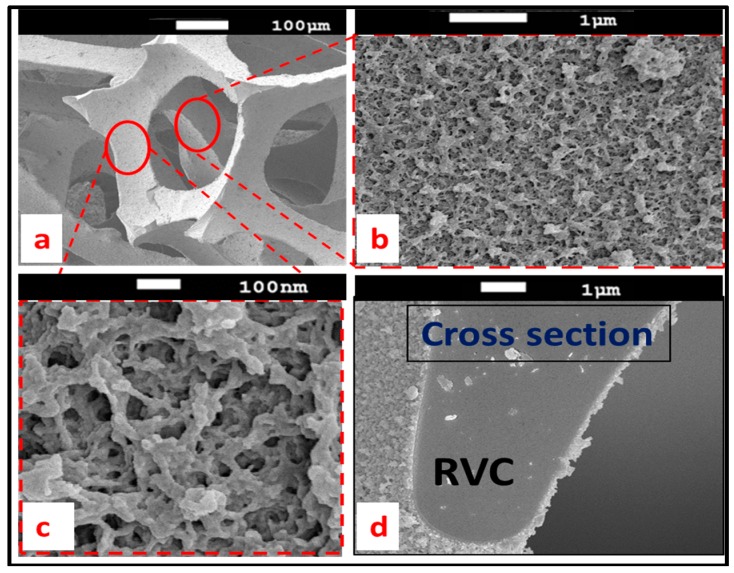
SEM of PEDOT coated RVC electrode for 10 min electropolymerization at 1.3 V: top surface (**a**–**c**); and cross-section (**d**).

Scanning electron microscopy (SEM) was used to collect information about PEDOT formation and its morphology along the RVC thickness. Figure 4 shows the SEM micrographs for 10 min electrodeposited PEDOT on RVC electrode. Figure 4a confirms that 10 min polymerization was enough to uniformly cover PEDOT on the RVC electrode and the average size of the pores of 60 ppi RVC is about 350 µm. It is clear that the surface of PEDOT coating is rough (Figure 4b) and highly porous (Figure 4c). This morphology can enhance the performance of PEDOT because of high conductivity, increased electrode surface area and specific capacitance, and macroporous structure of RVC electrode. Figure 4d shows the cross-section of a region of PEDOT/RVC composite electrode and it can be observed that the average thickness of PEDOT covering the RVC is around 280 nm.

**Figure 5:**
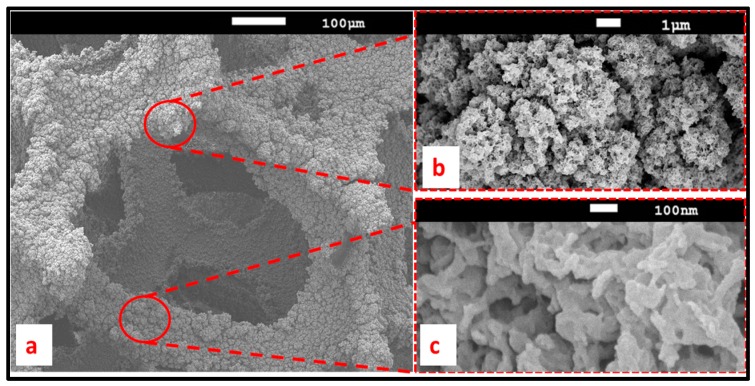
SEM of PEDOT coated RVC electrode for 120 min electropolymerization at 1.3 V, showing the top surface of (**a**) pore size; and (**b**,**c**) fuzzy like morphology.

Figure 5 shows a SEM of PEDOT coated RVC electrode prepared with 120 min electropolymerization of EDOT. It is very clear that the original pores of the RVC electrode about 350 µm size are not significantly affected by the PEDOT coating, and the average size of the composite electrode pores have become about 320 µm (Figure 5a). The PEDOT possesses an extraordinary fuzzy like and loose structure with honeycomb-like fine structure. The fine structure of PEDOT shows a flower-like morphology, consisting of numerous thin flakes with a number of fuzziness as seen in Figure 5b,c. The appearance of this structure is assumed to be related to the fast kinetics and will be useful for use as a capacitive deionization (CDI) electrode.

**Figure 6:**
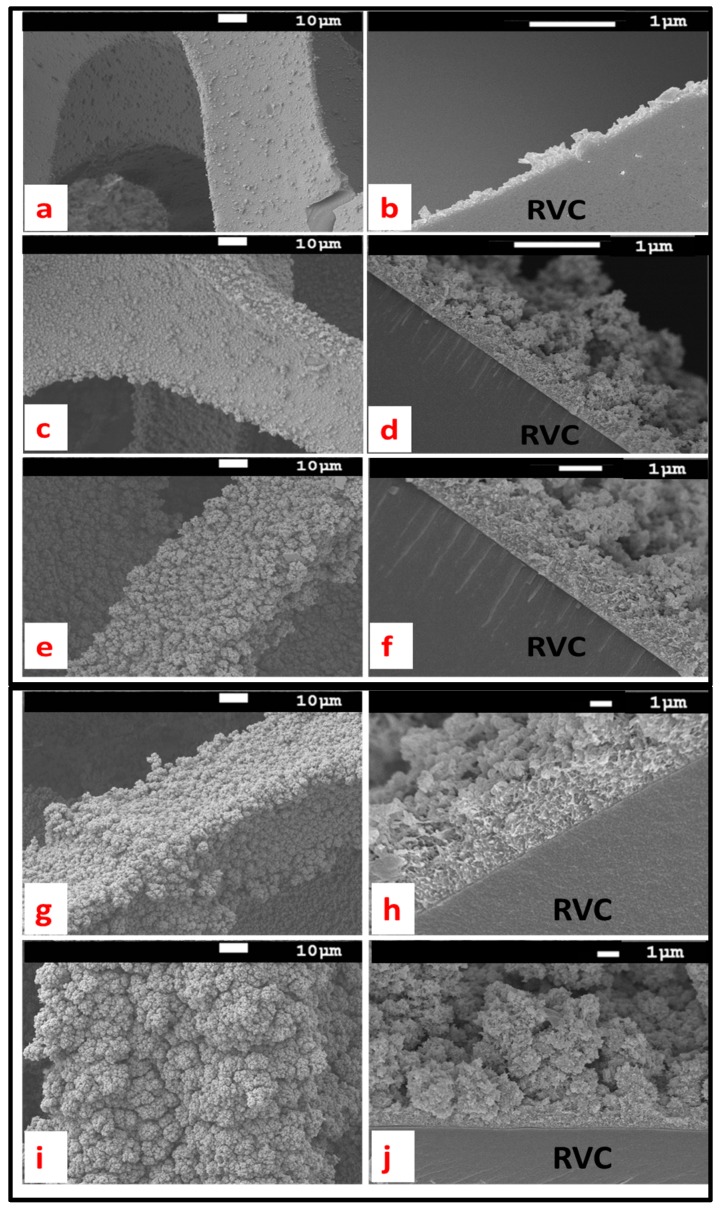
The top surface and cross-section of PEDOT coated RVC strut: for 10 min polymerization (**a**,**b**); for 20 min polymerization (**c**,**d**); for 50 min polymerization (**e**,**f**); for 70 min polymerization (**g**,**h**); and for 120 min polymerization (**i**,**j**).

The morphology of the electrosynthesized PEDOT on RVC electrode is affected by polymerization time. The PEDOT amount increased with increased polymerization time, as discussed above. Figure 6 shows SEM images of the strut of the RVC electrode after coating by PEDOT at various polymerization times. It is clear that PEDOT of relatively smoother surface covered all the strut of RVC after 10 min polymerization (Figure 6a) and the surface roughness was increased with increased polymerization time, as seen in Figure 6c,e,g,i. After 20 min polymerization, a globular structure of PEDOT started growing. Moreover, the globular structure of PEDOT increased upon increasing the polymerization time and also the fuzzy like morphology increased as a function of polymerization time. The thicknesses of PEDOT deposited on RVC electrodes were measured from the SEM image (Figure 6b,d,f,h,j). The thicknesses of PEDOT were 0.28, 0.61, 1.12, 2.53 and 4.64 µm at 10, 20, 50, 70 and 120 min electropolymerization, respectively.

### 3.6. Thermogravimetric Analysis

**Figure 7:**
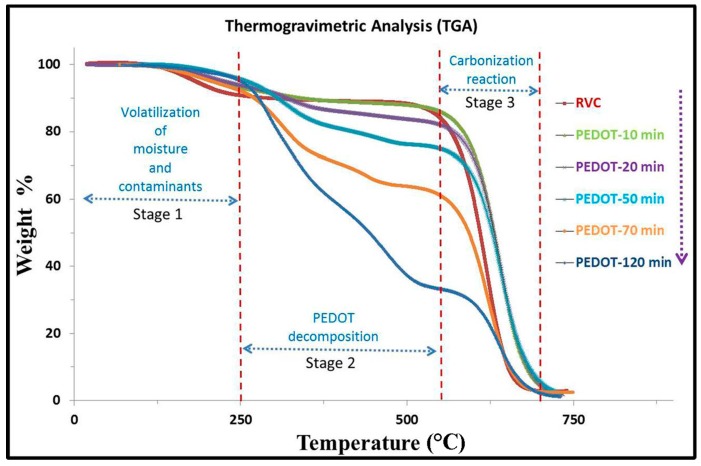
TGA curves of pure RVC electrode and various PEDOT/RVC composite electrodes.

The thermal stability and the amount of PEDOT coated on RVC electrodes were determined from the thermogravimetric analysis (TGA) curves which give the dependence of the weight loss of a sample as a function of temperature or time. Figure 7 shows TGA curves of pure RVC electrode and various PEDOT/RVC composite electrodes. It can be seen that the TGA curve of the RVC electrode exactly matches those reported in the literature [28,46], where 10% of the initial weight loss occurred between 25 and 250 °C, then it was stable and did not show a dramatic decomposition in the tested temperature range between 250 and 550 °C followed by a major weight loss between 550 and 700 °C. The TGA curve of the PEDOT/RVC composite electrode consisted of three stages [47,48]: volatilization, decomposition of PEDOT and carbonization reaction. All PEDOT/RVC composite electrodes followed the same behavior. The first 10% weight loss almost happened up to the temperature of 250 °C (Stage 1). This was probably due to desorption of moisture and contaminant. Furthermore, the major weight loss between 550 and 700 °C (Stage 3) was most likely due to carbonization by breaking chemical bonds such as C–H. It is clear that all composite electrodes followed a similar decomposition with temperature in Stage 2. From 250 °C a continuous degradation occurs until major decomposition occurred in the region between 350 and 550 °C. Therefore, in Stage 2, the amount of PEDOT in the various composite electrodes were calculated by loss in weight due to decomposition, and found to be 4%, 9%, 20%, 31% and 56% for 10, 20, 50, 70 and 120 min electropolymerization, respectively. In addition, almost no weight loss occurred after 700 °C.

### 3.7. Raman Spectroscopy

**Figure 8:**
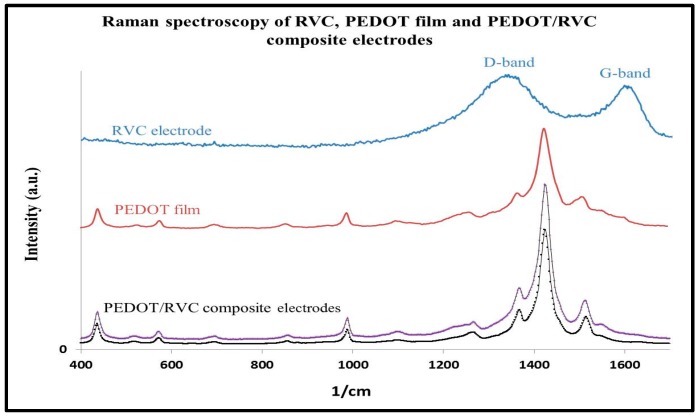
Raman spectra of PEDOT-ClO_4_ grown by chronoamperometry at 1.3 V in acetonitrile containing 0.01 M EDOT and 0.1 M LiClO_4_.

The Raman spectrum of the RVC substrate is shown in Figure 8 and is in keeping with published reports for RVC electrodes [49,50]. It has a typical two-band spectrum of disordered polycrystalline and noncrystalline graphitic carbons. The first band at 1360 cm^−1^ is called the D-band and the second band at 1600 cm^−1^ is called the G-band (graphitic) which is attributed to the graphite basal plane. The figure also shows the Raman spectra of RVC electrode after polymerizations that afford some information about the PEDOT structure. They confirm that the surface of RVC electrodes was coated by PEDOT. The principal assignments of the main bands have been made considering the data reported in the literature (Table 2) [34,51,52,53]. Raman spectra of composite electrodes have seven strong bands that dominate the spectrum which are related to the PEDOT vibrational spectrum. The most intense peak is at 1423 cm^−1^ which can be assigned to the symmetric C_α_=C_β_ (–O) stretching. Asymmetric C=C stretching shifts from 1509 cm^−1^ to 1507 cm^−1^ and at 1364 cm^−1^ appears a peak related to C_α_=C_β_ stretching. The asymmetric C_α_–C_α_– (inter-ring) stretching band is located at 1257 cm^−1^. The C–O–C deformation, which appears at 1152, 1120 and 1085 cm^−1^ peaks, combines as one peak at 1098 cm^−1^. Oxyethylene ring deformation peaks appear at 988 and 570 cm^−1^. In addition, the other peaks observed in the Raman spectrum of PEDOT coated RVC electrode are at 848 cm^−1^ (related to C–H bending of 2,3,5-trisubstituted thiophene due to α,α′ polymerization) and 685 cm^−1^ (symmetric C–S–C deformation). These results indicate that RVC electrodes do not affect the PEDOT structure.

### 3.8. Electrochemical Characterizations

Cyclic voltammetry was used to evaluate the electrochemical properties of the PEDOT/RVC composite electrodes. In this section, the effect of coating RVC by PEDOT on electrode capacitance, the effect of increasing scan rate on electron transfer and capacitance, and the stability of the electrode are considered. The specific capacitance of a PEDOT coating on RVC electrodes was calculated according to the following equations [54,55]:

*C*_volume_ = *Q*/(2 × *Z* × Δ*V*) (2)

*C*_mass_ = *Q*/(2 × *m* × Δ*V*) (3)

*C*_area_ = *Q*/(2 × *A* × Δ*V*) (4)

where *C*_volume_, *C*_mass_ and *C*_area_ are capacitance of electrode in term of F/cm^3^, F/g, and F/cm^2^, respectively; *Q* is charge (C); m is mass (g); *Z* is geometric volume (cm^3^); *A* is geometric area (cm^2^); and *V* is voltage (V).

### 3.9. Comparison between RVC before and after PEDOT Coating

**Figure 9:**
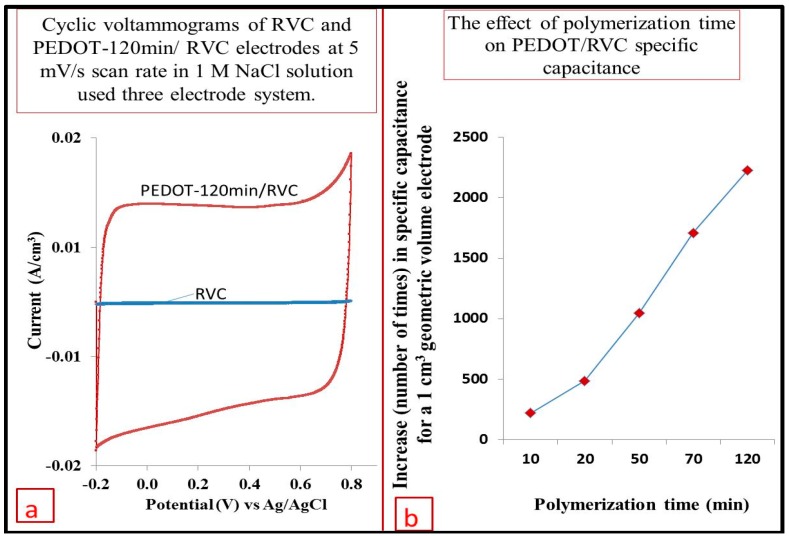
(**a**) Cyclic voltammograms of 1 cm^3^ bare RVC and same size of PEDOT-120 min/RVC composite electrode in 1 M NaCl using a scan rate of 5 mV/s and Ag/AgCl reference electrode; and (**b**) effect of increasing polymerization time of PEDOT on the specific capacitance of PEDOT/RVC electrodes.

Figure 9a shows the cyclic voltammograms of 1 cm^3^ RVC electrode and same electrode coated by PEDOT (PEDOT-120 min/RVC electrode) at the scan rate of 5 mV/s. It is clear that the CV curve of RVC compared with PEDOT coated electrode is very small. The capacitance of the RVC electrode in terms of geometric volume, calculated using Equation (2), is 0.002 F/cm^3^. The current of PEDOT-120 min/RVC composite electrode compared to a bare RVC electrode of same geometric volume has increased by a factor of 2230. This is related to the large surface area of PEDOT compared to a RVC electrode according to the Randle–Sevcik relationship [56]. Figure 9b shows the effect of increasing PEDOT in geometric volume of RVC on specific capacitance. It can be observed that the capacitance increased when polymerization time of PEDOT increased. The RVC geometric volume capacitance has increased by a factor of 220, 485, 1045 and 1710 at PEDOT coated RVC, where the PEDOT polymerization time was 10, 20, 50 and 70 min, respectively. The capacitance of PEDOT coated RVC electrodes at various polymerization time and various scan rates has been discussed more fully in the next section.

### 3.10. Effect of Increasing Scan Rate on the Electrode Capacitance

**Figure 10:**
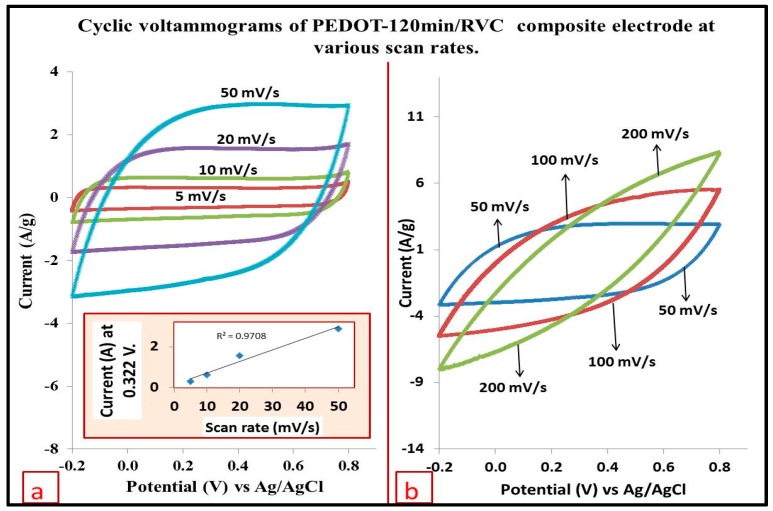
Cyclic voltammograms of PEDOT-120 min/RVC electrode at: (**a**) 5 to 50 mV/s scan rates; and (**b**) 50 to 200 mV/s scan rates in a three-electrode systems in 1 M NaCl solution.

In this study, the PEDOT-120 min/RVC composite electrode was also selected for the same previous reasons. Figure 10 shows the cyclic voltammograms of PEDOT-120 min/RVC composite electrode obtained at various scan rates of 5 to 200 mV/s. It can be noted that the shape of the CVs at scan rates up to 50 mV/s were nearly semi-rectangular and highly symmetrical (Figure 10a) which indicates an ideal behavior characteristic of double layered mechanism, fast charge/discharge process with insignificant ohmic resistance. The reason for its novel fast charge/discharge ability is that its granules are small and so encourage a large amount of meso-channels in the PEDOT/RVC composite, as shown in Figure 5, that can effectively reduce the diffusion length of ions (*L*) so as to reduce the diffusion time (*t*), which can be estimated as (*L*^2^/*D*) where *D* is the diffusion coefficient [57]. This shape helps to achieve a constant specific capacitance [58]. Furthermore, as the scan rate increased, the peak current also increased linearly (inset in Figure 10a). This result is similar to those published in the literature [59,60]. On the other hand, when the scan rate was increased above 50 mV/s, the curves were characterized by non-rectangular shapes (Figure 10b). This indicated resistance-like electrochemical behavior because the electrode is very porous, which hinders the migration of NaCl to the pores, and this becomes pronounced at increasing scan rates. This leads to a continuous decrease in the capacitance of electrodes with increasing scan rate; which is discussed in the next section.

### 3.11. Capacitance

**Figure 11:**
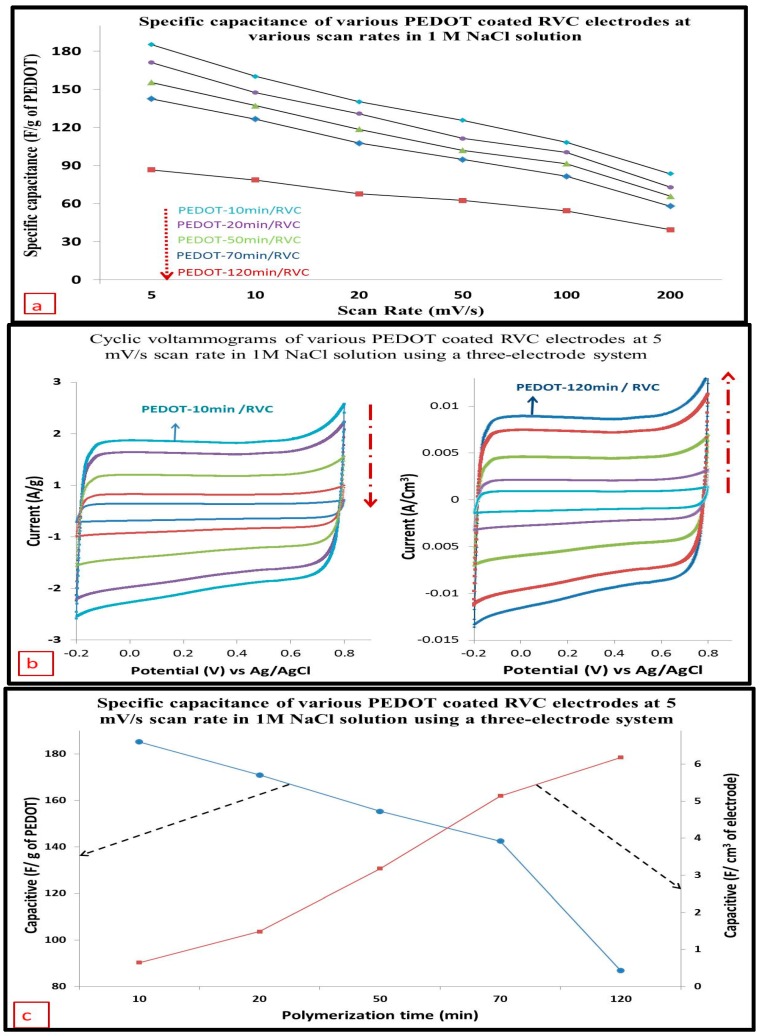
(**a**) The specific capacitance (F/g) of PEDOT/RVC electrodes at various scan rates; (**b**) cyclic voltammograms of PEDOT/RVC electrodes using current per gram of PEDOT and current per geometric volume of electrode at 5 mV/s scan rate; and (**c**) the specific capacitance of PEDOT/RVC electrodes in terms of F/g and F/cm^3^ at 5 mV/s scan rate. Electrolyte: 1 M NaCl solution. Potential range: between −0.2 and 0.8 V vs. Ag/AgCl using a three-electrode system.

The capacitive behavior of the PEDOT resulted mainly from electrochemical double-layer charging. The specific capacitances of PEDOT/RVC electrodes were calculated from the CV curves of PEDOT coated RVC electrodes using a three-electrode system at different scan rates of 5 to 200 mV/s, as shown in Figure 11a. It is observed that PEDOT has high capacitive behavior, and increasing the amount of PEDOT into the RVC electrode led to a decrease in the capacitance of the composite electrode. The specific capacitance of the PEDOT was significantly decreased from 185.29 F/g to 86.56 F/g when the polymerization time of PEDOT in the composite electrode was increased from 10 to 120 min, determined at a low scan rate of 5 mV/s. The value of specific capacitance of PEDOT at PEDOT-10 min/RVC electrode was very close to the value of theoretical specific capacitance (210 F/g) [61]. Furthermore, the specific capacitances of PEDOT coated RVC electrodes, in all electrodes, were found to be very high compared with other PEDOT studies [61,62,63] at low scan rate but its capacitance markedly decreased at high scan rates. The specific capacitance of the PEDOT-10 min/RVC was significantly decreased from 185.29 to 83.57 F/g as the potential scan rate was increased from 5 to 200 mV/s, as shown in Table 3. These results are in keeping with published reports for all cases of PEDOT [61,64,65]. It can be seen that the specific capacitance trend of all PEDOT electrodes decrease, when the scan rate was increased above 50 mV/s. It should be noted that the specific capacitance values in term of mass of all electrodes were calculated using Equation (3). As mentioned earlier, the aim of this study is to discuss the effect of increasing PEDOT amount in terms of geometric volume and area of the electrode on the capacitance results.

Figure 11b shows the comparison cyclic voltammograms for the same PEDOT/RVC electrodes using current per gram of PEDOT and current per geometric volume of electrode. It is very clear that current behavior in geometric volume unit was totally opposite to the current behavior in units of gram. Therefore, the capacitances per unit geometric volume of PEDOT/RVC electrodes were calculated using Equation (2) and are presented in the same Table 3 as F/cm^3^. The capacitance was 0.65 F/cm^3^ for the PEDOT-10 min/RVC and 6.18 F/cm^3^ for the PEDOT-120 min/RVC a low a scan rate of 5 mV/s. This indicates that the surface area of PEDOT coated in the RVC electrode was increased by increasing the amount of PEDOT, and the porous structure enhanced. Figure 11c shows that the trend of the capacitances in (F/cm^3^) was completely opposite to the direction of the trend for capacitance per gram.

Furthermore, the capacitance per unit geometric area was calculated using Equation (4) and are reported in the same Table 3 as F/cm^2^. It is expected that the capacitance (F/cm^2^) behavior of electrodes followed the capacitance (F/cm^3^) behavior. The capacitance has increased by a factor of 10 with increasing loading of PEDOT; that is, PEDOT-10 min/RVC electrode compared to PEDOT-120 min/RVC electrode.

### 3.12. Cycling Stability of PEDOT/RVC Electrodes

**Figure 12:**
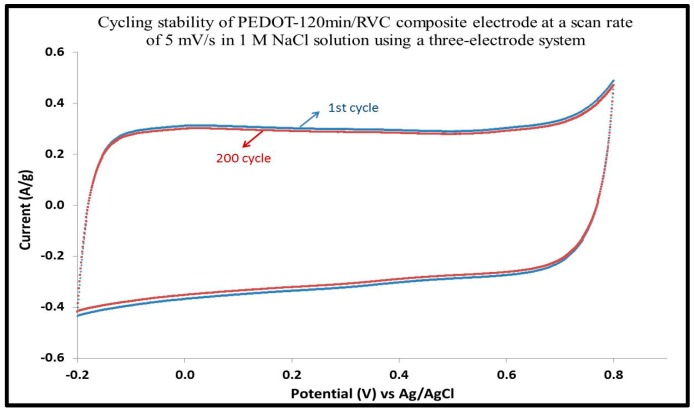
The electrochemical cycling stability of PEDOT-120 min/RVC electrode in 1 M NaCl solution recorded in the potential range between −0.2 and 1.0 V vs. Ag/AgCl using a three-electrode system at 5 mV/s scan rate for 200 cycles.

Stability is one of the most critical characteristics and electrodes with poor mechanical and electrochemical stabilities may lose their function. The electrochemical cycling performance of the PEDOT/RVC electrodes was investigated for 200 cycles at 5 mV/s, as shown in Figure 12. It can be observed that the CVs’ shape do not change much and are almost identical at the beginning and end of the stability test, as shown in other studies [66]. The PEDOT-120 min/RVC electrode shows quite good cycling stability and retains approximately 96% of its current density after 200 cycles. The loss may be due to the irreversible reactions of the PEDOT backbone, which represents a deterioration of the electrochemical reversibility [67]. The 3D mesoporous network structure can enable PEDOT to avoid shrinkage during continuous quick charging/discharging [68].

## 4. Conclusions

In this study, PEDOT has been successfully deposited by electropolymerization on RVC. The polymerization time decreases with the increase in electrode potential. SEM images confirm that 10 min polymerization was enough to uniformly cover PEDOT on the RVC electrode. The morphology of the PEDOT on RVC electrode is affected by polymerization time. The average pore size of RVC electrode is reduced marginally with the increase in polymerization time. The PEDOT amount, thickness, surface roughness, porosity, and globular structure on RVC electrode are increased with the increase in polymerization time. The surface of PEDOT coating was rough and highly porous, and loose with honeycomb-like fine structure, which shows a flower-like morphology, consisting of numerous thin flakes with numbers of macropores and micropores. This observed morphology can enhance the performance of PEDOT because of high conductivity, increased electrode surface area and specific capacitance, and macroporous structure of RVC electrode. TGA results show that the weight loss value of PEDOT increases with polymerization time. Raman spectroscopy and cyclic voltammetry suggest a strong interaction between the RVC and the backbone of the PEDOT chain. This improves the surface area and conductivity of the PEDOT/RVC composite electrode. It has been shown that the capacitance of PEDOT-120 min/RVC electrode has increased by a factor of 2230 compared to a bare RVC electrode. The capacitance of PEDOT was found to be very high compared with other PEDOT studies. The value of specific capacitances decreases with the increase in scan rate irrespective of polymerization time. The specific capacitance per unit mass is decreasing but the specific capacitance per unit volume and unit area is increasing with the increase in polymerization time irrespective of scan rate. The electrodes show good cyclic stability. Hence, the performance of RVC electrode has substantially increased by the electrodeposition of PEDOT on it. The results show that the PEDOT/RVC composites can be used as a capacitive deionization electrode.

## Figures and Tables

**Table 1:** Charge consumed during polymerization, polymerization time and mass of PEDOT coating on RVC electrode calculated by Equation (1).

Sample	Polymerization time (min)	Charge consumed (C)	Mass of PEDOT-ClO_4_ (mg)
PEDOT-10 min/RVC	10	20.64	13
PEDOT-20 min/RVC	20	46.04	29
PEDOT-50 min/RVC	50	112.71	71
PEDOT-70 min/RVC	70	185.73	117
PEDOT-120 min/RVC	120	380.98	240

**Table 2:** Calculated Raman band wavenumbers and vibrational assignments of PEDOT.

Description of the vibration	cm^−1^	Description of the vibration	cm^−1^
asym C= Cstr	1509	C–O–C def	1152, 1120 and 1085
CH_2_ Scissoring	1477	Oxyethylene ring def	988
Sym C_α_=C_β_(–O) str	1426	ClO_4_^–^	933
C_β_=C_β_ str	1365	C–H bending	806
C_α_=C_α_ str	1252	Sym C–S–C def	690
C_α_=C_α_ str	1236	Oxyethylene ring def	572

Key: str: stretching; def: deformation.

**Table 3:** Specific capacitance of PEDOT/RVC electrodes measured in various units at various scan rates.

**Composite electrode**	**PEDOT-10 min/RVC**	**PEDOT-20 min/RVC**	**PEDOT-50 min/RVC**	**PEDOT-70 min/RVC**	**PEDOT-120 min/RVC**
**Scan rate (mV/s)**	**Capacitance F/g**				
5	185.29	171.04	155.36	142.53	86.56
10	160.12	147.41	137.16	126.51	78.62
20	140.15	130.90	118.63	107.59	67.77
50	125.66	111.32	102.04	94.78	62.41
100	108.23	100.36	91.52	81.47	54.34
200	83.57	72.95	65.81	57.96	39.45
**Composite electrode**	**PEDOT-10 min/RVC**	**PEDOT-20 min/RVC**	**PEDOT-50 min/RVC**	**PEDOT-70 min/RVC**	**PEDOT-120 min/RVC**
**Scan rate (mV/s)**	**Capacitance F/cm^2^**				
5	0.08	0.19	0.41	0.66	0.80
10	0.07	0.17	0.36	0.59	0.73
20	0.06	0.15	0.31	0.50	0.63
50	0.06	0.12	0.27	0.44	0.58
100	0.05	0.11	0.24	0.38	0.50
200	0.04	0.08	0.17	0.27	0.36
**Composite electrode**	**PEDOT-10 min/RVC**	**PEDOT-20 min/RVC**	**PEDOT-50 min/RVC**	**PEDOT-70 min/RVC**	**PEDOT-120 min/RVC**
**Scan rate (mV/s)**	**Capacitance F/cm^3^**				
5	0.65	1.48	3.18	5.14	6.18
10	0.56	1.28	2.81	4.57	5.62
20	0.49	1.14	2.43	3.89	4.84
50	0.44	0.97	2.09	3.42	4.46
100	0.38	0.87	1.87	2.94	3.88
200	0.29	0.63	1.35	2.09	2.82

## Supplementary Materials

The following are available online at www.mdpi.com/2073-4360/9/5/157/s1, Figure S1: Scheme of oxidative polymerization mechanism of PEDOT.
