# Supplementary material for: A Strategy to Enhance the Electrode Performance of Novel Three-Dimensional PEDOT/RVC Composites by Electrochemical Deposition Method
Source: Polymers (Basel). 2017 Apr 28;9(5):157. doi: 10.3390/polym9050157 (PMC6431931; doi:10.3390/polym9050157)
Supplement: Supplementary file 1 [file polymers-09-00157-s001.pdf]

Supplementary Materials: A Strategy to Enhance the Electrode Performance of Novel Three-Dimensional PEDOT/RVC Composites by Electrochemical Deposition Method

Ali Aldalbahi, Mostafizur Rahaman and Mohammed Almoiqli

Polymerization Mechanism of PEDOT

The oxidation of EDOT to form PEDOT is similar to the oxidative polymerization of pyrrole [1]. The stepwise reaction mechanism of PEDOT is widely accepted for PEDOT oxidative polymerization [2–5] which is schematically shown in Figure S1. The first step generates a radical cation that has several resonance forms by oxidation of EDOT monomer. The second step is the combination of two radicals and subsequent deprotonation to form a neutral dimer. Substitution of the EDOT thiophene ring allows formation of new bonds. The dimer can be oxidized to form another positively charged radical that repeats the coupling and deprotonation steps with other monomeric or oligomeric cations. The alternating single and double bonds of the oligomers are π-conjugated, which delocalizes the electrons and decreases the oxidation potential. These stepwise reactions are repeated continuously to form the PEDOT polymer coated RVC electrode.

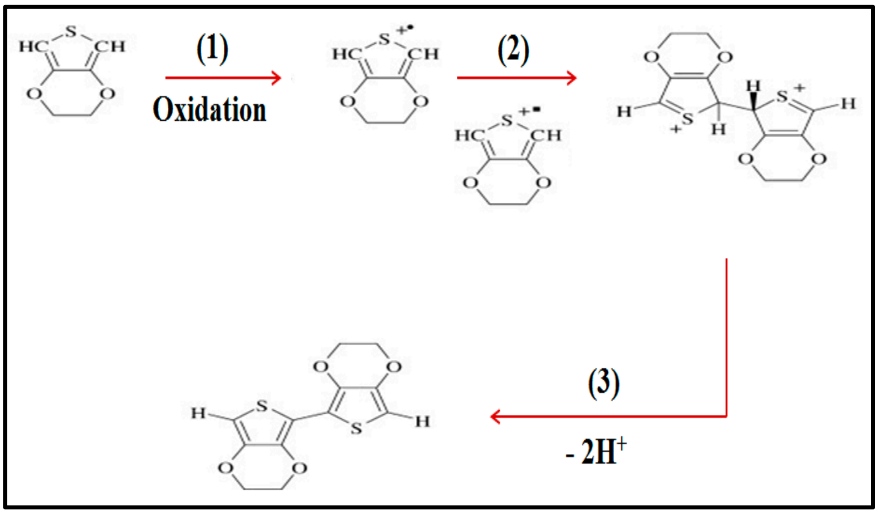

Figure S1. Scheme of oxidative polymerization mechanism of PEDOT.

References

1. Sadki, S.; Schottland, P.; Brodie, N.; Sabouraud, G. The mechanisms of pyrrole electropolymerization. *Chem. Soc. Rev.* **2000**, *29*, 283–293.
2. Kirchmeyer, S.; Reuter, K. Scientific importance, properties and growing applications of poly(3,4-ethylenedioxythiophene). *J. Mater. Chem.* **2005**, *15*, 2077–2088.
3. Lock, J.P.; Im, S.G.; Gleason, K.K. Oxidative Chemical Vapor Deposition of Electrically Conducting poly(3,4-ethylenedioxythiophene) films. *Macromolecules* **2006**, *39*, 5326–5329.
4. Im, S.G.; Olivetti, E.A.; Gleason, K.K. Systematic control of the electrical conductivity of poly(3,4-ethylenedioxythiophene) via oxidative chemical vapor deposition (oCVD). *Surf. Coat. Technol.* **2007**, *201*, 9406–9412.
5. Ha, Y.H.; Nikolov, N.; Pollack, S.K.; Mastrangelo, J.; Martin, B.D.; Shashidhar, R. Towards a transparent, highly conductive poly(3,4-ethylenedioxythiophene). *Adv. Funct. Mater.* **2004**, *14*, 615–622.
